# Recycling of Industrial Waste as Soil Binding Additives—Effects on Soil Mechanical and Hydraulic Properties during Its Stabilisation before Road Construction

**DOI:** 10.3390/ma17092000

**Published:** 2024-04-25

**Authors:** Witold Waciński, Ksawery Kuligowski, Małgorzata Olejarczyk, Marek Zając, Włodzimierz Urbaniak, Waldemar Cyske, Paweł Kazimierski, Robert Tylingo, Szymon Mania, Adam Cenian

**Affiliations:** 1 “WACIŃSKI” Construction Company, Długa 15 Str., 83-307 Kiełpino, Poland; 2 Institute of Fluid-Flow Machinery Polish Academy of Sciences, Fiszera 14 Str., 80-231 Gdańsk, Poland; 3 Faculty of Chemistry, Adam Mickiewicz University, Uniwersytetu Poznańskiego 8 Str., 61-614 Poznań, Poland; malgorzata.olejarczyk@amu.edu.pl (M.O.); wlodzimierz.urbaniak@amu.edu.pl (W.U.); 4 Pracownia Drogowa Waldemar Cyske, Borówkowa Str. 51, 83-010 Rotmanka, Poland; 5 Department of Food Chemistry, Technology and Biotechnology, Faculty of Chemistry, Gdańsk University of Technology, Narutowiczas Str. 11/12, 80-233 Gdańsk, Poland; robertt@pg.edu.pl (R.T.); szymon.mania@pg.edu.pl (S.M.)

**Keywords:** road construction, soil stabilization, soil binders, compressive strength, soil capillary forces, frost resistance, waste additives, industrial waste, pyrolytic wax, emulsions

## Abstract

To improve the in situ soil stabilization, different chemical additives are used (ion exchange compounds, additives based on H_2_SO_4_ or vinyl polymers, and organic additives using lignosulfonates). One interesting alternative is the production of additives from various waste materials. The extensive testing of waste-based blends with soil was performed; the mechanical (unconfined compressive strength (UCS)) and hydraulic (capillary rise, water absorption, and frost resistance (FR)) soil properties were measured. The optimization process led to obtaining additive compositions ensuring high strength and sealing properties: by-pass ash from the ceramics industry, waste H_2_SO_4_, pyrolytic waxes/oils from waste mixed plastics, waste tires and HDPE, and emulsion from chewing gum waste. For sandy soil, the following additives were the most promising: emulsion from pyrolytic wax (EPW) from waste PE foil (WPEF) with the addition of waste H_2_SO_4_, pyrolytic-oil emulsion from waste tires, EPW from waste mixed plastics with the addition of “by-pass” waste ash and NaOH, EPW from WPEF with the addition of NaOH, and EPW from WPEF reaching up to 93% FR, a 79.6% 7-day UCS increase, and a 27.6% of 28-day UCS increase. For clay: EPW from WPEF with the addition of NaOH, EPW from WPEF with the addition of waste H_2_SO_4_, and solely EPW from WPEF reaching up to 7.5% FR, an 80.7% 7-day UCS increase, and a 119.1% 28-day UCS increase.

## 1. Introduction

Soil stabilization is a common process for almost all road projects. All types of soil stabilization can be divided into two basic groups, i.e., mechanical stabilization and chemical stabilization. In mechanical stabilization, the classification of the soil is changed by mixing it with soils of different classes. This way, a compacted soil mass can be obtained. Chemical stabilization, on the other hand, involves modifying soil properties by adding chemically active materials [1]. In the following article, we will focus on chemical stabilization using waste materials. The use of waste as a building material for soil stabilization is a new area in the construction industry [2].

Modification of native soil characteristics during road construction (if the soil does not meet the requirements of frost resistance, bearing capacity, etc.), with waste that does not have a negative impact on the ecosystem, is beneficial for the environment. On the one hand, the use of natural resources is minimized, which reduces the environmental degradation of the place where these aggregates are obtained. On the other hand, the use of waste materials gives them functional properties and enables the desired soil properties to be achieved, while reducing the amount of waste going to landfills [3].

Classic stabilizers that chemically affect the native soil and strengthen it are lime, cement, bitumen, fly ash, and others [4,5]. Of all the soil stabilization techniques, improvement by adding cement and lime is one of the most widespread and widely studied in recent decades. Research on the stabilization of calcareous and cement soils has resulted in comprehensive theoretical knowledge on the mechanisms of stabilization accumulated over many years. In addition, the effectiveness of stabilizing calcareous and cement soils has been investigated and confirmed by many authors [6,7,8,9,10,11,12,13]. Umar and Linii found in their research that the use of marble powder as a limestone material improves the geotechnical properties of clayey material. They showed that the optimal marble powder content for maximizing engineering effects is approximately 60% dry matter. CBR capacity ranges from 10.43% to 22.94% for unsoaked conditions and 4.68% to 12.46% for soaked conditions with 60% marble powder [14]. An experimental study made by Gallaego-Quintana to analyze the mechanical properties of a soil stabilized with ordinary Portland cement (OPC) under a sustainable approach consisting of a significant substitution of OPC for sugarcane bagasse ash (SCBA) to reduce the quantity of cement used in the stabilization shows that the 25% partial replacement of OPC by SCBA shows the best performance in its maximum dry density, reaching a compressive strength and California bearing ratio significantly close to the soil values with 100% OPC; this could imply, in practical terms, a reduction in cement consumption [15].

Another way to improve soil cohesiveness parameters is through the addition of a hydrophobic agent. Hydrophilizing anionic chemical solutions that are currently available on the market give unsatisfactory results because they do not change the hydrophilic character of the soil. This is because the anionic components of these substances are not attracted to the negatively charged minerals contained in the soil [16].

Many studies also show that plastic waste serves to improve the parameters of the native soil. Combining it with the soil showed some improvement in terms of soil strength, but even so, the potential of this waste has not been fully assessed for the different types and forms of plastic waste combined with the natural soil substrate in road construction [17,18,19,20].

The geotechnical and mechanical properties of soils stabilized with waste, such as biomass ash from electricity generation, along with small amounts of silica-based nanotechnology stabilizers, are being studied. The results obtained so far suggest a potential reduction in the use of traditional binders by incorporating such by-products while maintaining soil properties and even improving properties through the use of nanosized additives [21]. Stevulova et al. have shown that the use of cement-bypass fly ash with alkali activation results in a cementitious material with good mechanical properties [22]. Recently, various polysaccharide-based biopolymers have been used to improve the hydromechanical properties of soils. Xanthan gum, starch, chitosan, cottage cheese, glucan, agar gum, gellan gum, guar gum, and sodium alginate are conventional biopolymers in geotechnical engineering [23,24,25].

The research of Song et al. [25] shows that polyol prepolymers can effectively improve the mechanical properties of clay soil. The existence of lattice membrane structures induced by polyol prepolymer/water reactions linked soil particles together, which significantly improved the cohesion of clay soil, and the angle of internal friction was maintained at a relatively stable level. Waciński et al., in their patent application no. P.438697, also described a dust-based hydraulic binder with a sodium hydroxide activated bypass [26]. The use of alkaline materials allows geopolymers to be formed by creating a three-dimensional network of interconnected molecules resembling conventional mineral binders [27]. 

Bauxite is a typical alkaline waste used in the stabilization process [28,29,30]. Alzhanova et al. showed that the alkaline properties of bauxite residues decrease with time after use: cation convertibility and sodium exchange capacity decrease [2]. In addition to stabilizing the soil, bauxite also has properties that stabilize heavy metal and fluorine pollution [31].

Another stabilization approach is soil hydrophobic (so-called artificial hydrophobic soils), which are new geomaterials with great potential for engineering applications due to their low affinity for water. For saline soils, hydrophilization of such soils can mitigate soil salinization and subsequent engineering disasters such as salt expansion and chloride corrosion. Hydrophobic agents such as organosilanes, fatty acids, and waxes are commonly used to induce soil hydrophobicity [32,33,34,35]. 

Compared to natural sands, wax-coated sands are hydrophobic and exhibit excellent, although not yet fully understood, mechanical and hydraulic properties. Bardet et al. [35] showed that a wax content of up to 6% can triple the permeability and double the compressibility. These models show that the increase in permeability is related to the aggregation of smaller sand particles due to the stickiness of the wax, which generates larger gaps. Smaller particles attach to other particles and make the sand thicker. The wax coating also softens the contact between the sand particles and makes the wax-coated sand more compressible.

In the current study, the authors investigated more than 600 test scenarios using waste-derived materials for improving soil mechanical (unconfined compressive strength (UCS)) and hydraulic (capillary rise, water absorption, frost resistance (FR)) properties in clay and sand. The novelty of the study is based on seeking an optimal scenario for:

1. The best strengthening and sealing soil-stabilizing additives;
2. The utilization of waste materials in a safe manner without posing a pollution risk to the environment.

The authors chose waste-based materials that are highly abundant and accessible from various branches of the industry, energy, and waste sectors. Such prepared and optimized unique mixtures and doses were not found elsewhere in the reviewed literature. The novelty also comes from the treatment of the waste, e.g., via a combination of thermal and chemical methods such as pyrolysis, emulsification, and sulphonating.

The aim of the research was to develop an optimal method of supporting the stabilization of both cohesive and non-cohesive soils, which is an addition to the traditional hydraulic binder (BV cement). The main idea was to use waste materials, especially those for which no alternative use has yet been found.

## 2. Materials and Methods

**Figure 1:**
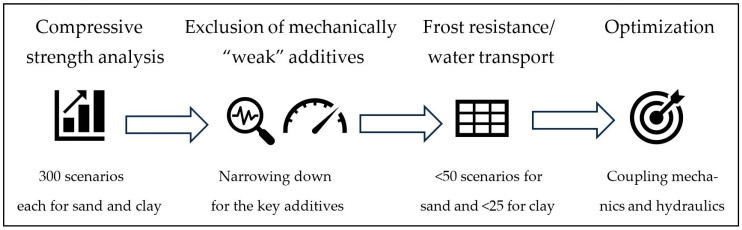
The concept of the methodology covering mechanical and hydraulic properties examination followed by optimization.

In accordance with the assumptions adopted above, tests were carried out for 5% cement content in relation to the soil mass. Each time, 8 kg of soil has been used. To obtain greater reliability, each type of soil was sampled from the same source before each test. The Figure 1 below shows the concept of the methodology applied herein.

### 2.1. Materials—Commercial Soil Stabilizing Additives

Road construction costs have boomed considerably over the last few years; hence, there is a need to look for possible means of reducing them. This could be partially achieved by replacing commonly available polymer-based commercial stabilizers with alternative waste-derived recycled materials. The authors preselected a wide variety of waste-derived materials and examined some of them, as shown in Table 1. These are: cement bypass dust which is a by-product of the ceramics industry (ash), waste sulfuric acid (acid), pyrolytic wax from waste mixed plastic (wax), and chewing gum waste.

The benchmark for examining alternative stabilization additives was to compare them with those available on the market. Before starting the first tests, several weeks of extensive market research was carried out on the available soil stabilization additives. Below are descriptions of several selected additives commercially available on the market used in soil stabilization.

1. Geosta K-1 constitutes ion exchange agents that have a beneficial effect on cement hydration. Basically, it is a mixture of salts, i.e., chlorides of magnesium, sodium, potassium, calcium, and iron; potassium carbonate and iron; aluminum; and sodium sulfates. The pioneer of this type of additive is the Dutch company Geo-Products B.V. (Maastricht, The Netherlands), which has been offering a loose ion exchange agent under the trade name GEOSTA K-1 for many years, including on the Polish market [36].
2. ROADBOND EN 1™ (Saint Andrews, MB, Canada) stabilizer causes clay to release weakly ionized water molecules from the clay matrix and replaces the water with strongly ionized sulfate radicals. The exchange is permanent and takes place at normal pH levels. As in lime stabilization, metal hydrates are formed, which help increase the strength of the clay, and by leaving the clay matrix intact, the permeability is significantly reduced. When mixed with base material and compatible in-place material, ROADBOND EN 1™ stabilizer dissolves the mineral salts and natural cementitious properties of the soil. Mixing the soil disperses the dissolved material into the void spaces between the soil grains, where it cures and crystallizes. The re-crystallized mineral salts and natural cements form an effective bond that results in improved strength, load-bearing capacity, and durability. The replacement of weakly ionized water molecules with strongly ionized sulfate radicals, along with the increased dry density, make the treated soil more resistant to water penetration. This reduces shrink-swell potential along with freeze–thaw damage [37].
3. ETONIS^®^ 1400 S is a polymer dispersion for soil stabilization. When ETONIS^®^ 1400 S is applied to the soil, the product forms polymer bridges between the soil or aggregate particles and hydrated cement particles. ETONIS^®^ 1400 S is responsible for the formation of a flexible and robust network that is durable and water-resistant [38]. ROKAmin SRK8P4 is a specialized cationic surfactant. The product is a quaternized, ethoxylated, and oxypropylated fatty amine of animal origin. The product is available in the form of a clear, viscous liquid with a maximum color of 130 on the iodine scale. ROKAmin SRK8P4 dissolves well in hot water as well as in organic solvents such as chloroform. Supplied by Wacker Chemie AG Headquarters, Hanns-Seidel Platz 4, 81737 Munich, Germany.
4. PROROAD™, supplied by Phoenix Distribution (Mornington, VIC, Australia), is a sealing and stabilizing fluid (2 separate components) supplied in concentrated form in 1000 L IBC containers or 200- and 120-L barrels. The final product is non-toxic, environmentally safe, and easy to use. There is no requirement to use special construction teams due to the application of the product. The specific gravity for Proroad is 1.06 and 1.4 for Proroad Waterproof.
5. TEQUAT LC90i is a cationic surfactant. It is obtained by the condensation of fatty acids of animal origin and triethanolamine, followed by quaternization. The product is offered in isopropanol, in the form of a soft paste with a color ranging from white to yellow. Tequat LC90i contains 90% of the active substance with the INCI name: Dihydrogenated Tallowethyl Hydroxyethylmonium Methosulfate and Ditallowethyl Hydroxyethylmonium Methosulfate. The product forms dispersions in water. Supplied by PCC Group, Sienkiewicza 4, 56-120 Brzeg Dolny, Poland [39].

### 2.2. Materials—Waste-Derived Additives

During the research, the authors used individually prepared soil additives made from waste, which are equivalent to commercially available additives.

1. Based on CBPD (cement bypass dust), waste ash from the ceramic industry, the so-called ash from the brickworks bypass, is an alternative to the popularly used commercial binder GEOSTA (constituting a mixture of salts, i.e., chlorides of magnesium, sodium, potassium, calcium, and iron, potassium carbonate and iron; aluminum; and sodium sulfates) described in the text below as ash.
2. Sulfuric acid of waste origin, which is an alternative to the popularly used Roadbond EN-1 soil stabilizer. It fundamentally alters the ability of clay to hold adsorbed water, which is water held by electrical attraction.
3. Pyrolytic oils and waxes from waste plastics, described in the text below as polyolefin emulsions, based on products formed during the thermal destruction of polyolefins, made of:
  ⚬ pure HDPE (High Density PolyEthylene);
  ⚬ waste mixed plastics;
  ⚬ waste tires;
  ⚬ waste PE foil.
4. Emulsion from chewing gum waste.

Many of the different types of waste turned out to have a positive impact not only on the strength but also on the sealing of the stabilized soil. The waste was sourced from various industries, e.g., the food industry, ceramics industry, chemical industry, cosmetics industry, energy sector, municipal waste processing plants, etc.

### 2.3. Mixture Preparation

Before starting the mechanical tests, a detailed scenario was developed regarding the tested additive and its amount. For each scenario, separate samples of 8 kg of soil (cohesive or non-cohesive) were measured using a scale. The samples of soil, cement, and additive were taken to the mixer, where they were added in the following order: soil, cement, water, and additive. Mixing took no less than 5 min to obtain a homogeneous consistency. The mixture was then removed from the mixer and delivered to the compactor.

### 2.4. Sample Preparation for Mechanical Tests

After preparing the mixture, it was transferred to the next station and placed in molds, where it was then compacted in an automatic compactor in three cycles of 25 blows in accordance with the PN-EN 13286-50 standard [40]. The completed samples were placed in foil to retain moisture, and after 24 h, they were removed from the mold and weighed. Then, all samples were placed in containers with moist sand for further tests.

### 2.5. Compressive Strength Test

The compressive strength test was carried out on an automatic hydraulic press (Marshall PM-CBM comp) in accordance with the EN 13286-50 standard [40]. In accordance with the adopted assumptions, samples were tested for durability after 7 or 28 days of curing. At least 3 samples were used to conduct the strength test. The press results were given in kN. This result was written down and then converted into MPa. The area of the base of the sample is 25π cm^2^ ≈ 78.5 cm^2^ = 0.00785 m^2^. To convert kN/m^2^ to MPa, the formula 1 kN/m^2^ = 0.001 MPa was used, i.e., for example, 1 kN of pressure on a hydraulic PM-CBM press corresponded to approximately 0.127 MPa. The final result for each additive was always the arithmetic average obtained from 3 samples.

### 2.6. Capillary Rise Test

Soil hydraulic properties denote the potential of the additive-amended soil to prevent water penetration from the bottom upwards by means of capillary forces and from the surroundings inwards during total immersion. The sealing properties of the mixtures were tested using proprietary tests developed by the company. This test consisted of two stages: a capillary rise test and a water absorption test. The result of the test was the measurement of the capillary rise/water absorption coefficient (%). This coefficient was calculated from the formula:

(1) $$WN=\left(1-\frac{m_{w}}{m_{s}}\right)\times 100\%$$

where:

- *WN*—Capillary rise/absorption coefficient (%);
- *m_w_*—mass of the soaked sample (via capillary rise or immersion) (g);
- *m_s_*—mass of dried sample (g).

The test was carried out on 7-day-old samples. A minimum of 2 samples were used for each scenario. After collecting the samples from the moist sand, they were placed in a dryer and dried overnight at a temperature of 50 °C. After this time, the dried samples were marked and weighed on a scale.

**Figure 2:**
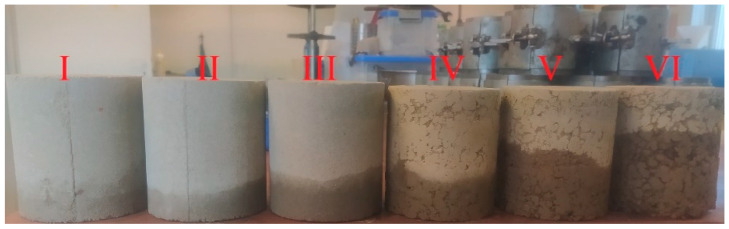
Example view of the samples during the capillary rise test. Samples I–VI represent soil samples after 4 h capillary rise test (sand on I–III and clay on IV–VI) after various treatments.

After weighing, the samples were tested for capillary rise. This test involved placing the dried samples in a container flooded with water to a height of 2 cm. After 4 h, the samples were removed, photographed, and weighed. Then, the mass of the dry sample was subtracted from the result of the weighed sample, and the mass of absorbed water was obtained. The obtained results were substituted into the formula, and the water absorption via capillary rise coefficient was determined. Figure 2 presents the view of random soil samples pictured in the 4 h capillary rise increasing order.

### 2.7. Water Absorption Test

After the capillary rise test, the water absorption test was carried out. The samples were then placed back in the container and flooded with water until they were completely covered. After 20 h, the samples were taken out and weighed again. The mass of the dry sample was subtracted from the obtained result, and the mass of water absorbed by the entire sample was obtained. All the results were introduced into Formula (1) to determine the water absorption coefficient. After weighing, the samples were additionally subjected to strength tests.

### 2.8. Frost Resistance Test

The frost resistance test was carried out on 28-day-old samples in accordance with the EN 13286 41 standard [41] in accordance with WT 5. It involves examining the durability of samples after 14 cycles of freezing and thawing. The study was carried out together with a durability test for 28-day samples, the results of which were the reference point. A minimum of 3 samples (usually 4) were used to conduct the test. The test began by placing the samples in water for approximately 7 h to soak them. Then, the soaked samples were weighed and subjected to 14 alternating freezing and thawing cycles.

Freezing was carried out by placing the samples in a freezer at a temperature of approximately −20 °C for at least 4 h. Then, the samples were placed in containers with water at a temperature of approximately +20 °C to thaw them. After a minimum of 2 h, the samples were removed from the water, ending one cycle, and placed back in the freezer, starting the next cycle. After the 14th cycle, the thawed samples were weighed again to check for weight loss and subjected to strength tests. According to the standard, samples should not lose more than 5% of their weight and should retain 60% of their strength. Here, the frost resistance is defined as the % change in sample compressive strength (after 28 days of curing) after the 14th cycle of freezing.

### 2.9. Soil Stabilization Standards

Pavement design is based on the analysis of the following parameters:

Road traffic, soil (its properties), and the materials from which we want to build the surface. In relation to the soil, the following parameters are the most important:

- Load capacity;
- Bursting.

Ground heave can be assessed in two ways:

- By analyzing the type of soil, the content of fine particles, the sand index, and water conditions (groundwater depth, embankment height, excavation depth);
- By testing the load-bearing capacity of the substrate in the most humid or saturated conditions—the CBR index should be determined.

Based on the CBR or soil and water conditions, the subgrade load-bearing group is determined, from G1 to G4. The load-bearing capacity group of the subgrade is one of the basic input data points for designing pavement structures. This procedure is the basis for designing pavement structures in accordance with Order of the General Director for National Roads and Motorways No. 31 GDDKiA of 16 June 2014, which introduces the Catalog of Typical Flexible and Semi-rigid Pavement Structures developed by Gdańsk University of Technology, 2014. In our studies, we aimed at achieving at least 2 MPa of compressive strength, which is in line with the Polish WT-5 standard information [42].

**Figure 3:**
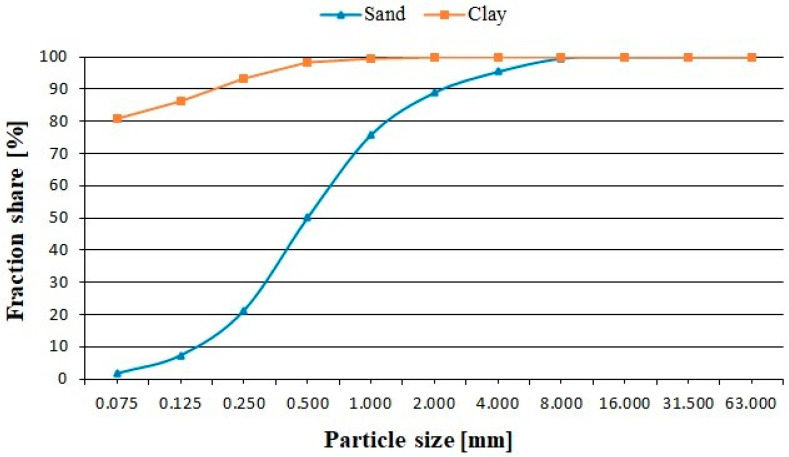
Particle size distribution of the soils used in the study: sand and clay.

Basic soil properties expressed as particle size distributions are presented below on Figure 3.

## 3. Results

### 3.1. Single Additions—Compressive Strength

The tests were carried out separately for clay and for sand (control scenarios with only cement in the amount of 5% by weight). Then the additives were tested. They were the basis for developing original compositions for hydraulic binders. The tests are presented below.

#### 3.1.1. Commercial Additives

Table 2 shows the results of the mechanical properties of soil after using commercial additives. It appears that only ROADBOND EN-1 for both soils and ETONIS 1400S for clay have some positive effects on increasing the compressive strength; however, these increments are moderate and vary across samples. The standard control treatment with only 5% cement (for clay average from 17 samples and for sand average from 19 samples) led to compressive strengths of ~0.93 and ~1.04 MPa for clay and sandy soil, respectively.

#### 3.1.2. CBPD “By-Pass” Ash and Sulphuric Acid

The results of the average values of compressive strength for binders based on the addition of substitutes from CBPD “by-pass” ash and waste sulfuric acid are presented in Table 3. The addition was from 10 to 200 g of the CBPD-waste ash (Geosta substitute) and from 0.6 to 1.2 mL of the waste sulfuric acid (Roaddbond EN-1 substitute) with 5% cement and 8 kg of soil.

As shown in Table 3, the addition of ash slightly increased the compressive strength of both clay and sand if compared with the control treatment, whereas the addition of acid had either no effect (clay) or a negative effect (sand).

#### 3.1.3. Pyrolytic Waxes

The results of the average compressive strength values for binders based on the addition of emulsions from pyrolytic waxes (EPW) are summarized in Table 4. The addition was from 10 to 40 g with 5% cement and 8 kg of soil.

The results indicate a positive effect of the use of wax emulsions, with the best results observed for waste PE foil emulsions (37% increase for clay and 55% increase for sand), while waste mixed plastics EPW were slightly less effective (33% for clay and 42% for sand). In contrast, the effect of using a wax emulsion made from HDPE turned out to be two times better on clay than on sand. Any differences are probably due to the inevitable impurities contained in thermally processed waste polyolefins. The good news is that waste mixed plastics, which are much more polluted than waste PE foil, do not lead to a significant reduction in the bearing capacity of the soil compared to a cleaner material such as waste PE foil. Standard deviations reached approximately 15–20%.

Two treatments with EPW from WMP and EPW from WPEF reached the minimum limit for stabilized subbase which is 1.25 MPa in our climate zone.

#### 3.1.4. Emulsions from Chewing Gum Waste

As shown in Table 5, the addition of chewing gum waste in the form of emulsion slightly increased the compressive strength of clay, whereas for sand, it even reduced the compressive strength of the material.

### 3.2. Hybrid Additions—Compressive Strength

Due to the fact that the addition of the ash alone causes a slight improvement in compressive strength (5.7% and 11.1% for clay and sand, respectively) and the use of the waste sulfuric acid (Roundbond EN-1 substitute) does not affect the strength (Table 1), it was decided to enrich them with the addition of an emulsion of pyrolytic waxes, expecting a hybrid effect of additives from both previously defined groups.

The results of the average compressive strength values for binders based on hybrids, i.e., the addition of substitutes (ash and acid) and the addition of emulsions from pyrolytic waxes are summarized below (Table 6). The addition of substitutes was from 10 to 200 g of “by-pass” waste ash and from 0.6 to 1.2 mL of waste sulfuric acid, and the emulsion was from 10 to 40 g with 5% cement and 8 kg of soil.

#### 3.2.1. CBPD “By-Pass” Waste Ash and Sulphuric Acid with the Addition of EPW

**Figure 4:**
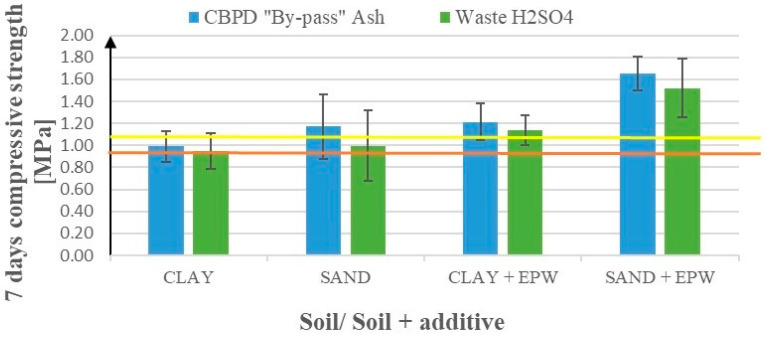
Effects of the addition of substitutes on compressive strength (first 4 bars) and the addition of substitutes enriched with pyrolytic wax emulsions (next 4 bars). The yellow line represents the control value (average compressive strength for samples treated using only cement without additives) for sand and the brown line for clay.

As shown on Table 6 and Figure 4 below the addition of EPW significantly improved the compressive strength of soils where only substitutes were used (Table 2). Thus, the strength on clay increased by almost 30% and 22% (for the Geosta and EN-1 substitutes, respectively), and on sand by as much as 57% and 44% (for the Geosta and EN-1 substitutes, respectively). Standard deviations were greater for sand results (up to 30%) and smaller for clay results (up to 15%), which would mean that sand showed greater heterogeneity than clay and, in practice, that the influence of the above-mentioned additives and their hybrids will be more visible in this soil.

Both treatments with EPW presented in Table 6 reached the minimum limit for a stabilized subbase, which is 1.25 MPa in sandy soil in our climate zone.

#### 3.2.2. EPW from Polyolefins with the Addition of Substitutes of the Commercial Stabilizer (“By-Pass” Waste Ash and Waste Sulfuric Acid)

Despite the good results of the increase in compressive strength after using an emulsion made of pyrolytic waxes from various waste polyolefins, the results for binders, where the wax emulsion was also enriched with a substitute (Geosta or EN-1), are presented separately below in Table 7.

The effect of this addition compared to the use of EPW itself turned out to be small for the HDPE emulsion (from 31 to 33% for clay), slightly more significant for the EPW from waste mixed plastic (WMP) (42 to 54% for sand), and decreased for the EPW from waste PE foil (WPEF)—from 37% to 22% for clay and from 55% to 44% for sand. Therefore, taking into account the results from Table 1 and Table 2, it is suggested to use EPW as the main additive and, depending on the soil and situation, to use emulsion hybrids with substitutes, which gave the highest results for the combination of emulsions with Geosta substitute (up to 30% on clay and 57% on sand). Standard deviations reached approximately 15–20%.

Two treatments with EPW from WMP + “by-pass” waste ash and EPW from WPEF + waste H_2_SO_4_ reached the minimum limit for a stabilized subbase, which is 1.25 MPa in our climate zone.

#### 3.2.3. EPW from Polyolefins with the Addition of Emulsion from Chewing Gum Waste or NaOH

The compressive strength results for hybrids in the form of EPW from waste polyolefins (HDPE and WMP) + chewing gum emulsion and EPW from waste PE foil + NaOH are listed in Table 8. The best results using chewing gum waste were achieved for HDPE emulsion, i.e., up to 43% growth on clay, up to 24% on sand, and up to 35% on clay when using WMP emulsion. The addition of NaOH to the EPW from WPEF had no effect on the results on clay, but for sandy soil, it increased the compressive strength by up to almost 70%.

One treatment with EPW from HDPE + chewing gum waste reached the minimum limit for a stabilized subbase, which is 1.25 MPa, and another with EPW from WPEF + NaOH reached the minimum limit for a stabilized base for light traffic in our climate zone.

#### 3.2.4. Emulsions from Chewing Gum Waste with the Addition of EPW from WMP or CBPD “By-Pass” Waste Ash from the Ceramic Industry

**Figure 5:**
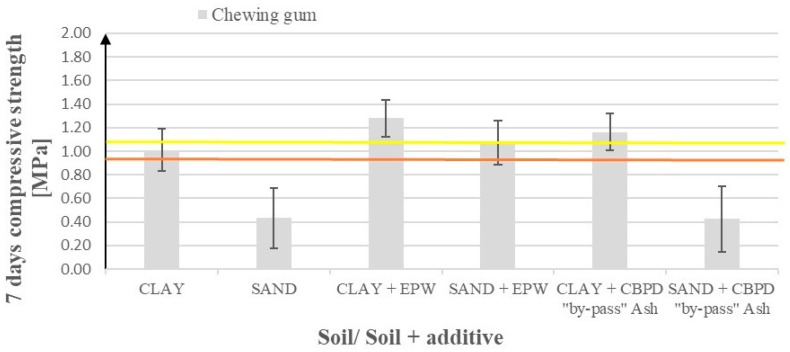
Effect of the addition of emulsion from chewing gum waste (first 2 bars) and additionally with (1) EPW WMP (next 2 bars) and (2) Geosta substitute (last 2 bars) on soil compressive strength. The yellow line represents the base value (compressive strength using only cement without additives) for sand and the brown line for clay.

As shown in Table 9 and its graphical interpretation on Figure 5, the effect of using chewing gum emulsion (both alone as well as with the addition of other substances) on sandy soil compressive strengths is negative (in relation to the standard addition of cement). When clay is enriched with chewing gum emulsion in a mixture with the EPW from WMP or ‘by-pass’ ash, a 37% or 24% increase in strength is observed, respectively. Standard deviations for increases reached 15–20% and even up to 50% for decreases.

### 3.3. Soil Water Absorption and Frost Resistance—Introductory Investigations of Response to Hybrid Additions

Besides compressive strengths, the hydraulic properties of the samples, like water absorption and frost resistance, are of great importance for road construction technologies. The introductory investigation has already shown that for sandy soil, the addition of 10–50% (by weight in relation to cement) of a 1:1 water emulsion of pyrolytic wax from WPEF could ensure maximum frost resistance of 70–93% (35,6% for the control sample with 5% cement addition), which is closely related to the low water absorption (0.35–8.00%) of these samples.

To further improve sample parameters, the addition of NaOH was proposed. The introductory test proved that the addition of NaOH to EPW from WMP causes an increase in compressive strength, i.e., from 8.6% to 33% (after 7 days) and to 24% (after 28 days of curing). A further increase is achieved after the addition of “by-pass” waste ash enriched with NaOH, as discussed in Section 3.4.

Only EPW from WMP (1.63 MPa), CBPD “by-pass” ash + EPW from WMP (1.66 MPa), and EPW from WPEF + NaOH (1.79 MPa) exceeded the 1.6 MPa, which is close to the minimum limit for stabilized bases for light traffic in our climate zone (1.75 MPa). Few of the treatments reached the minimum limit for a stabilized subbase, which is 1.25 MPa.

Table 10 shows the results for control scenarios where only 5% cement was used. This is helpful especially when referring to the relative compressive strength values expressed in % in Table 11 and onwards.

### 3.4. Soil Compressive Strength, Water Absorption, and Frost Resistance after Hybrid Additions

#### 3.4.1. The Best Sealing Materials—Sorted by Frost Resistance

To select the best additives, one should take into account not only mechanical properties (like compressive strengths) but also hydrological ones (like frost resistance). Table 11 presents results related to the studied materials sorted (from left to right) by frost resistance value. Only results with a higher frost resistance than 48.47% are presented. The control value of resistance for the scenario with only the 5% cement addition was 35.6%.

In general, hybrids with EPW considerably increased the frost resistance (even over 93%) but kept the compressive strength increase (after 28 days of curing) at low levels (below 24.71%). However, waste H_2_SO_4_ and “by-pass” waste ash showed much better 7-day compressive strengths (over 37.46% increase), with the frost resistance not that significant (ca. 48.5–69.5%), although much greater than 35.6% for the control sample with 5% of cement. It should be pointed out that addition of EPW from WMP enriched using “by-pass” waste ash with NaOH gives probably the best results here, i.e., a combination of rather high frost resistance (57%) with relatively low water absorption (3.5% after 4 h and 6.3% after 24 h) as well as compressive strengths that increase up to 70% after 7 days of curing (2.07 MPa) and up to a further 25% after 28 days (2.53 Mpa).

#### 3.4.2. The Best Sealing Materials—Sorted by the Capillary Rise

Here, the studied materials were sorted by the capillary rise value (water absorption over 4 h). Only results better (lower) than 1.1% are presented (the control value is 6% for the binder with only cement). Table 12 below shows the best scenarios when taking into account the minimal capillary rise, preventing soils from absorbing too much water.

In general, all considered additives significantly reduced the capillary force (even more than 10 times); addition of sodium stearate, purification by sulfonation, or adding soap and cream production wastes significantly increased the compressive strength after 7 days of curing (from 8% to 41%).

Table 13 refers to the modification of the mechanical and hydraulic characteristics of clay soil for the selected additives. Only additives leading to a capillary rise value lower than 7% are presented (the control value is 4.24% for standard sealing with a 5% cement addition). In general, all additives (except the first one) somewhat reduced the capillary force, but not as drastically as for sandy soil.

The best results for sealing properties (both capillary rise and water absorption) and compressive strengths after 28 days of curing were achieved for the additives of EPW from WPEF enriched with NaOH, which provided exceptionally high compressive strengths (119% after 28 days of curing). When enriched with waste H_2_SO_4_, the parameters are slightly weaker, except for compressive strengths after 7 days of curing.

#### 3.4.3. The Best Strengthening Materials—Chosen Taking into Account Compressive Strength after 7 Days

Table 14 presents the hydraulic and mechanical properties of sandy soil with the best strengthening additives (such as EPW from waste polyolefins enriched with waste H_2_SO_4_, NaOH, “by-pass” waste ash, cream, and powder) sorted by the compression strength after 7 days of curing.

In general, all additives considered here increased the compressive strength after 7 days of curing significantly (up to 142%), maintaining capillary force below 3.5% and water absorption below 8%.

Table 15 presents the hydraulic and mechanical properties of clay soil with the best strengthening additives. In general, the treatments increased the compressive strength (up to 119%); unfortunately, the capillary force increased often above the 4.24% control value. Only the addition of NaOH and the commercial additive TequatLC90i improves the performance of EPW from WPEF, both in terms of clay sealing and strengthening properties.

#### 3.4.4. Best Strengthening Materials—Chosen Taking into Account Compressive Strength after 28 Days

Table 16 presents the best of the studied additives, which were selected taking into account their compression strength after 28 days of curing.

In general, most of the studied additions increased the compressive strength after 28 days of curing (up to 27.59%); the capillary force and water absorption were mostly under the control value. Only EPW from waste PE foil expressed a negative 28-day compressive strength, but further additions of NaOH, “by-pass” waste ash, and NaOH, as well as waste H_2_SO_4_, further improved this value. All scenarios showed very high frost resistance.

Some studies also evaluated the benefits of using different waste ashes, such as fly ash (FA), rice husk ash (RHA), and face masks (FM), as additives for soil stabilization [43]. When the soil was modified with three additives, the highest strength gain occurred when the soil was modified with 10% of RHA, 15% of FA, and 0.3% of FM. Among all the additives, 10% RHA produced the highest strength. Table 17 shows both mechanical and hydraulic properties of the best additives used in clay taking into account 28 days compressive strength.

All results from the studied materials were sorted by compression strength after 28 days. In general, only two treatments increased the compressive strength (changes of 64.42% and 119%) while maintaining the capillary force under the desired 4.24% control value. With both, the addition of EPW from WPEF and its further enrichment by the addition of commercial ProRoad Waterproof worsened both sealing and 28-day strengthening properties.

The results presented above are in line with the published ones. The addition of alkaline activators such as NaOH commonly utilized in geopolymeric soil stabilization was also noticed elsewhere [44,45,46]. For example, researchers from Brazil [44] found that the maximum lateritic gravelly 7-day soil compressive strength reached 2.2 MPa when applying sugarcane bagasse ash with 7M NaOH, regardless of the ash proportion (3–10%). Another study [47] focused on the application of bottom ash, marble dust, and tire rubber powder (TRP) to replace cement to stabilize problematic clay soils. It was reported that 2.5% TRP replacement of cement at low density seems to be the optimum dosage to provide the best performance (unconfined compressive strength of 4.5 MPa for 7% cement addition, initial stiffness, and accumulated loss of mass).

## 4. Discussion

The effects of waste additions on the mechanical and hydraulic properties of sandy soil and clay were investigated, and the results are discussed below.

### 4.1. Effects of Waste-Based Additives on Compressive Strength

**Figure 6:**
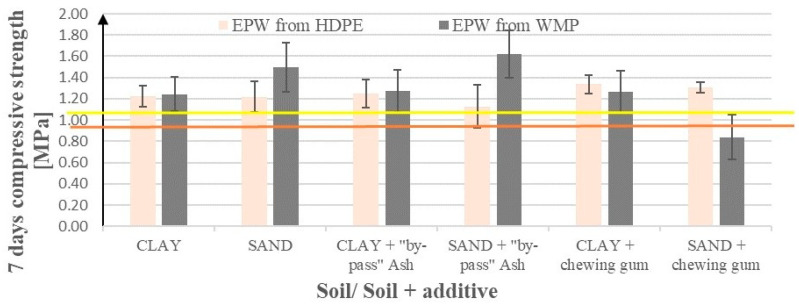
Soil compressive strengths after 7 days of curing following the addition of emulsions from pyrolytic waxes (HDPE or WMP, called RDF here—first four bars), additionally enriched with the Geosta substitute (next four bars), or with emulsion from chewing gum waste (last four bars). The yellow line represents the base value (compressive strength using the addition of only 5% of cement) for sand and the brown line for clay.

Figure 6 presents compressive strengths after 7 days of curing for modified sandy soil and clay samples after the addition of waste products. These products include emulsions from pyrolytic wax (EPW) resulting from the pyrolysis of polyolefins (HDPE and waste mixed plastics (WMP)) and waste from the production of chewing gum, as well as waste ash from the ceramic industry (a substitute for Geosta commercial products). Figure 6 shows the effects of EPW from WPEF (waste PE foil) addition enriched with NaOH or waste H_2_SO_4_.

The use of pyrolytic waxes from polyolefins increased the compressive strength of the clay samples by around 10–40% regardless of the type of polyolefins or additional material used (except for chewing gum with WMP, which worsened the mechanical properties—see Figure 6).

For sand, increases are more varied (5–80%), with the highest values for EPW from WPEF emulsion and its hybrids (Figure 6), i.e., EPW from WMP emulsion and the Geosta substitute, EPW from WPEF enriched with the EN-1 substitute (H_2_SO_4_), and EPW from WPEF enriched with NaOH. In contrast, the use of the chewing gum emulsion in a hybrid with EPW from WMP remains irrelevant to the compressive strength. The standard deviations for both soils were generally insignificant, except for the chewing gum scenarios, where they reached 15–25%.

In general, the use of EPW from WMP increases the compressive strength of both sand and clay soils more than EPW from HDPE (except in the case of hybrids with chewing gum emulsion, where the tendency is opposite).

**Figure 7:**
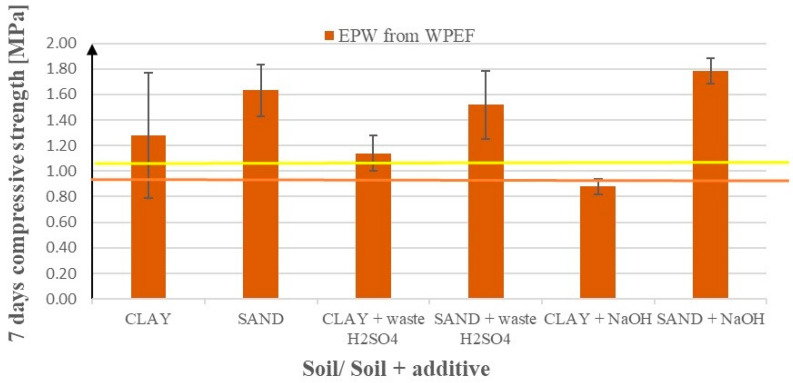
Soil compressive strengths after 7 days of curing after the addition of EPW from WPEF (the first two bars) additionally enriched with waste H_2_SO_4_—the EN-1 substitute (the next two bars), and NaOH (the last two bars), on the soil compressive strength. The yellow line represents the base value (average compressive strength using only cement without additives) for sand and the brown line for clay.

According to Figure 7, the EPW from the WPEF hybrid with NaOH exhibits a positive effect on the compressive strengths of sand and has no effect on clay. If we exchange NaOH with waste H_2_SO_4_, the effects for sand are almost as good as for NaOH, and for clay, they are slightly better.

In another study [48], an unconventional approach has been introduced by utilizing brick kiln dust and pond ash as novel materials replacing traditional soil stabilizers, such as lime and cement, and waste materials, such as fly ash and rice husk ash. The combined effect of brick kiln dust and pond ash had an exponential influence on maximum dry density and optimal moisture content. In addition, soil stabilization with a 30% content of brick kiln dust (optimum content) increases the California bearing ratio (CBR) by 76–143% for different soils, whereas an addition of 30% pond ash (optimum content) increases the CBR by 69–96% for different soils. Researchers from Brazil and Australia [46] concluded that sugarcane bagasse ash and eggshell hydrated lime present a clay compressive strength for a high-density binder above 2.1 MPa, which is the threshold value defined for a stabilized base for medium traffic.

### 4.2. Effects of Waste-Based Additives on Hydraulic Properties (Water Transport and Frost Resistance)

As the mechanical and hydraulic properties are both important for the road construction industry, the correlations between soil mechanical and hydraulic parameters were studied.

**Figure 8:**
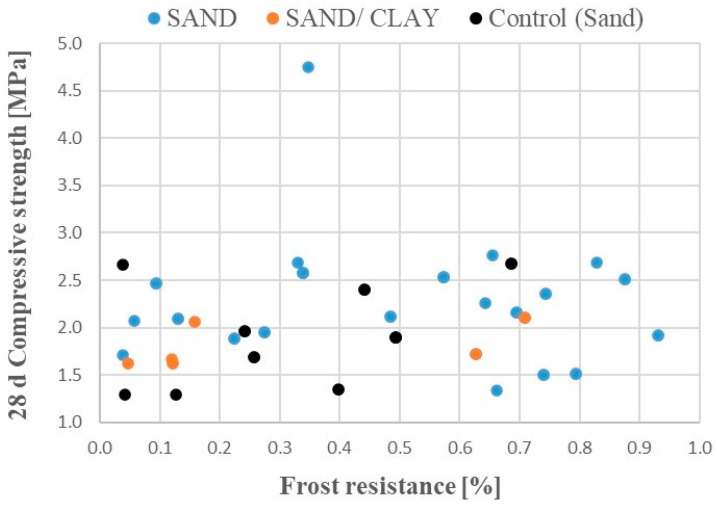
Sandy and clay soil compressive strengths after 28 days of sample curing as a function of frost resistance; the control is sandy soil with a 5% cement addition.

Soil stabilized prior to road construction should possess good mechanical and hydraulic properties. To study the relation between these properties, a hypothesis was formulated that the more empty spaces, cracks, and thin pores, the stronger the capillary forces and hence the lower the frost resistance. At the same time, compression strength in such highly perforated soil should be lower. Therefore, both relations should somehow be inversely proportional. However, Figure 8 presents a lack of a clear correlation between compression strengths and frost resistance.

**Figure 9:**
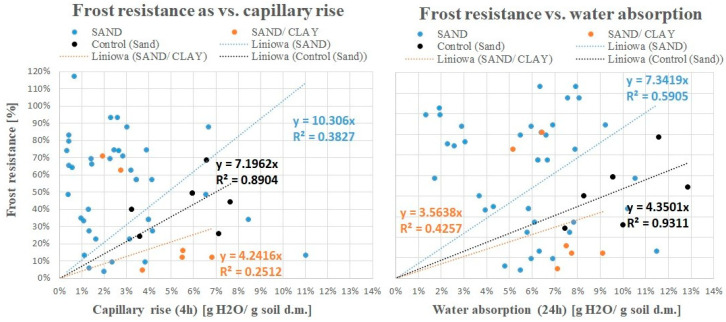
Frost resistance as a function of capillary rise (**left figure**) and as a function of water absorption (**right figure**).

Figure 9 below (left part) presents the correlation between frost resistance and capillary rise. Both properties are strongly correlated only in the control samples (with 5% cement addition)—correlation coefficient (0.8904). In this case, the frost resistance is easy to calculate (without the need to conduct 14-cycle frost tests) by multiplying the capillary rise value by a factor of 7.1962. There is no clear relationship for mixtures with sealing additives, or there is not enough data to demonstrate this relationship (correlation coefficients of 0.3827 for sand and 0.2512 for sand and clay). This is probably due to the high heterogeneity of the samples, additives, and different compositions.

In the case of the correlation between frost resistance and water absorption (Figure 9, right part), a linear correlation was found again only for control samples with a high correlation coefficient (0.9311). Frost resistance is easily calculated by multiplying the water absorption value (% of the mass of the water absorbed in relation to the initial dry mass of the soil) by the factor 4.3501. There is no clear relationship between samples and sealing additives, or there is not enough data to demonstrate this relationship (correlation coefficients of 0.3827 for sand and 0.4257 for sand and clay).

### 4.3. Optimized Strengthening and Sealing Materials—Sorted by Optimization Parameter

To select the best materials for use in soil stabilization both in terms of bearing capacity (increasing compressive strength) and in terms of tightness/sealing and preventing road damage by frost-thaw cycles (capillary rise/water absorption affecting frost resistance) presented above, an artificial “optimization parameter” was proposed.

First, the compressive strength after 28 days of curing was standardized, relating it to 1 kg of soil, because different additives and their amounts affect the bulk density of the sample, which translates into strength. Hence, a new set of data was generated, the so-called relative strength related to soil mass [MPa/1 kg of soil]. Then, the capillary rise parameter was standardized, defined as the capillary rise after 4 h in % of absorbed water divided by the above-mentioned relative strength in MPa/kg of soil. As a result, an “optimization parameter” is obtained, which combines both sealing properties (capillary rise after 4 h) and mechanical properties (compressive strength after 28 days of curing).

Table 18 presents the results for sandy soil sorted by the “optimization parameter”. The lower the parameter, the better the strengthening and sealing properties of the sample with additives. The lowest values of the optimization parameter (below 70) were achieved for the following additives: EPW from WPEF, EPW from WPEF + waste H_2_SO_4_, waste tire pyrolytic oil emulsion, WMP + “by-pass” waste ash + NaOH, and EPW from WPEF + NaOH.

Table 19 presents results for clay sorted by the “optimization parameter”. The lowest values of the optimization parameter (below 300) were achieved for the following additives: EPW from WPEF + NaOH, EPW from WPEF + waste H_2_SO_4_, and solely EPW from WPEF. Unfortunately for the last scenario, the sealing properties (capillary rise and water absorption) were above the control values.

A research group from Iran [4] concluded that the use of 15% ceramic waste powder and 6M NaOH increased the natural soil compression strength from 0.080 to 1.2 MPa (for 7 days of curing) and to 2.22 MPa (for 28 days of curing). In parallel, the soil flexibility expressed by the failure strain increased from 2.31% to 5.45%. This backs up the synergistic effects altering the mechanical and hydraulic properties of the “by-pass” waste ash and NaOH used in the current study.

Linking soil water absorption (hydraulic properties influencing sealing) and compaction (mechanical properties influencing strength) in clay soil was also found elsewhere [49], where the study investigated the factors influencing the water absorption characteristics of waste-based additives, including paper sludge ash (PSA), palm kernel shell ash (PKSA), rice husk ash (RHA), coal fly ash, hemihydrate gypsum, limestone powder, and basalt rock powder (BRP). The water absorption of PSA, PKSA, and RHA suggests that the addition of these waste-based additives to clays with high water content improves their compaction properties early in curing.

## 5. Conclusions

A wide variety of waste-based materials were tested as additives to in situ soil/cement mixtures for both sealing and strengthening properties to stabilize soil. They act as hydraulic binders and could effectively substitute commercial polymers, reducing the price of road stabilization and contributing to the circular economy. The extensive research presented above shows both scenarios where single additives are used and those where hybrids or combinations of selected additives are used. The drawback of using such additives is their lack of homogeneity. This could imply a thorough quality check prior to use.

From the groups of additives selected above, the best additive scenarios were selected in terms of frost resistance and compressive strength. As a result of the optimization process presented above, the following individual additives ensuring the best strengthening and sealing properties are proposed:

- For SAND: EPW from WPEF + waste H_2_SO_4_ (69.5% FR, 45.6% 7-day UCS increase, 8.8% 28-day UCS increase) waste tire pyrolytic oil emulsion (74.3% FR, 17.5% 7-day UCS increase, 18.9% 28-day UCS increase), EPW from WMP + “by-pass” waste ash + NaOH (57.3% FR, 79.6% 7-day UCS increase, 27.6% 28-day UCS increase), EPW from WPEF + NaOH (87.6% FR, 40.9% 7-day UCS increase, 26.6% 28-day UCS increase), and EPW from WPEF (93% FR, 15.1% 7-day UCS increase);
- For CLAY: EPW from WPEF + NaOH (7.5% FR, 40.8% 7-day UCS increase, 119.1% 28-day UCS increase), EPW from WPEF + waste H_2_SO_4_ (2.8% FR, 80.7% 7-day UCS increase, 64.4% 28-day UCS increase), and solely EPW from WPEF (6.9% FR, 6.9% 28-day UCS increase).

A fairly good correlation between frost resistance and capillary rise for the control samples (with 5% cement addition) was found (R^2^ = 0.8904); however, no clear relationship between these parameters was found for mixtures with waste-based sealing additives. Real-scale testing is recommended before making a final decision.

Only soil treated with EPW from WMP (1.63 MPa), CBPD “by-pass” ash + EPW from WMP (1.66 MPa), and EPW from WPEF + NaOH (1.79 MPa) exceeded the 1.6 MPa, which is close to the minimum limit for stabilized base for light traffic in our climate zones (1.75 MPa). Few of the treatments reached the minimum limit for a stabilized subbase which is 1.25 MPa.

## 6. Patents

1. “Hydraulic sealing binder for cohesive soils and the method of its production and connection with the native cohesive soil”, No. P.438697, on behalf of Construction Company WACIŃSKI Witold Waciński
2. “Sealing additive for hydraulic binder for non-cohesive soils and grained native soils, method of its production, and connection with native soil”, No. P.444266, on behalf of Construction Company WACIŃSKI Witold Waciński

## Figures and Tables

**Table 1:** The list of waste-based materials chosen for the tests.

Waste-Based Material Tested	Dosages Used/8 kg of Soil and 5% Cement	National Potential [t/year]	Commercial Equivalent
1. Pyrolytic wax from mixed plastic waste, emulsion (EPW from WMP)	<700 g	4–5 M	ROKAmin ^1^/ Tequat LC90i ^2^
2. CBPD “by-pass” waste ash from the ceramic industry	10–200 g	30,000	Geosta ^3^
3. Emulsion from chewing gum waste	10–40 g	12,000	ROKAmin/ Tequat LC90i
4. Waste tire pyrolytic oil (WTPO), emulsion	20–140 g	Not in production	ROKAmin SRK8P4/Tequat LC90i
5. Pyrolytic wax from HDPE, emulsion (EPW from HDPE)	20–100 g	Not in production	ROKAmin SRK8P4/Tequat LC90i
6. Pyrolytic wax from waste PE foil, emulsion (EPW from WPEF)	20–700 g	Not in production	ROKAmin SRK8P4/Tequat LC90i
7. Waste sulfuric acid with limonene	<420 g	No data	Roadbond EN-1 ^4^
8. Waste cream emulsion from the cosmetics industry	10–60 g	120	ROKAmin/ Tequat LC90i
9. Waste powder, including soda (sodium bicarbonate), from the cosmetics industry	40–140 g	60	ROKAmin/ Tequat LC90i

^1, 2^ PCC Group manufactured, Brzeg Dolny, Poland; ^3^ Geo-Products B.V. manufactured, Maastricht, The Netherlands; ^4^ Fort Distributors Ltd. manufactured, Saint Andrews, MB, Canada.

**Table 2:** Average soil compressive strength values for substitutes of commercial stabilizers.

Additive	Geosta K-1		ROADBOND EN-1		ETONIS 1400S	
Soil	Clay	Sand	Clay	Sand	Clay	Sand
Samples	11	7	2	1	3	5
MPa (7 d)	0.47	0.45	1.10	1.17	0.96	0.85
SD/2	0.13	0.18	0.21	0	0.18	0.21
Change (%) ^1^	−49.56%	−56.85%	17.27%	11.34%	−25%:+52%	−19.72%

^1^ Refers to the average control treatment where only 5% cement was used (for clay average from 17 samples and for sand average from 19 samples); SD/2—standard error.

**Table 3:** Average soil compressive strength values for substitutes for commercial stabilizers.

Additive	“By-Pass” Waste Ash		Waste H_2_SO_4_	
Soil	Clay	Sand	Clay	Sand
Samples	63	63	24	23
MPa (7 d)	0.99	1.17	0.95	0.99
SD/2	0.14	0.29	0.16	0.32
Change (%) ^1^	5.67%	11.08%	1.36%	−5.63%

^1^ Refers to the average control treatment where only 5% cement was used (for clay average from 17 samples and for sand average from 19 samples).

**Table 4:** Average soil compressive strength values with the addition of EPW from waste polyolefins (HDPE, mixed plastics—WMP, PE foil—WPEF).

Additive	EPW from HDPE		EPW from WMP		EPW from WPEF	
Soil	Clay	Sand	Clay	Sand	Clay	Sand
Samples	7	8	13	14	17	20
MPa (7 d)	1.23	1.22	1.24	1.49	1.28	1.63
SD/2	0.10	0.15	0.16	0.23	0.49	0.20
Change (%) ^1^	30.77%	15.67%	32.75%	41.75%	36.77%	54.84%

^1^ Refers to the average control treatment where only 5% cement was used (for clay average from 17 samples and for sand average from 19 samples).

**Table 5:** Average soil compressive strength values with the addition of emulsions from chewing gum waste.

Additive	Chewing Gum Waste	
Soil	Clay	Sand
Samples	48	32
MPa (7 d)	1.01	0.43
SD/2	0.18	0.26
Change (%)	7.92%	−58.99%

**Table 6:** Average soil compressive strength values for substitutes of commercial stabilizers with the addition of EPW from waste mixed polyolefins.

Additive	CBPD “By-Pass” Ash + EPW		Waste H_2_SO_4_ + EPW	
Soil	Clay	Sand	Clay	Sand
Samples	14	14	8	9
MPa (7 d)	1.21	1.66	1.14	1.52
SD/2	0.16	0.15	0.13	0.27
Change (%) ^1^	29.67%	57.12%	21.87%	44.14%

^1^ Refers to the average control treatment where only 5% cement was used (for clay average from 17 samples and for sand average from 19 samples).

**Table 7:** Average soil compressive strength values with the addition of EPW from polyolefins (HDPE, waste mixed plastics (WMP), and waste PE foil) enriched with substitutes of commercial stabilizers.

Additive	HDPE EPW + “By-Pass” Waste Ash		EPW from WMP + “By-Pass” Waste Ash		EPW from WPEF + Waste H_2_SO_4_	
Soil	Clay	Sand	Clay	Sand	Clay	Sand
Samples	3	4	8	10	9	9
MPa (7 d)	1.25	1.13	1.27	1.62	1.14	1.52
SD/2	0.13	0.20	0.20	0.22	0.14	0.27
Change (%) ^1^	33.25%	6.96%	35.74%	53.76%	21.69%	44.14%

^1^ Refers to the average control treatment where only 5% cement was used (for clay average from 17 samples and for sand average from 19 samples).

**Table 8:** Average soil compressive strength values with the addition of emulsions from pyrolytic waxes from waste polyolefins (HDPE, WMP, PE foil) enriched with the emulsion from chewing gum waste and NaOH.

Additive	EPW from HDPE + Chewing Gum Waste		EPW from WMP + Chewing Gum Waste		EPW from WPEF + NaOH	
Soil	Clay	Sand	Clay	Sand	Clay	Sand
Samples	4	4	8	4	3	7
MPa (7 d)	1.34	1.31	1.26	0.84	0.88	1.79
SD/2	0.09	0.05	0.20	0.21	0.06	0.10
Change (%) ^1^	42.70%	24.20%	34.91%	−20.65%	−6.15%	69.35%

^1^ Refers to the average control treatment where only 5% cement was used (for clay average from 17 samples and for sand average from 19 samples).

**Table 9:** Effects of the addition of an emulsion made from chewing-gum waste alone and with the addition of (1) EPW from WMP or (2) a CBPD “by-pass” ash on the compressive strengths of clay and sandy soil.

Additive	Chewing Gum		Chewing Gum + EPW WMP		Chewing Gum + “By-Pass” Ash	
Soil	Clay	Sand	Clay	Sand	Clay	Sand
Samples	48	32	13	8	22	12
MPa (7 d)	1.01	0.43	1.28	1.07	1.16	0.43
SD/2	0.18	0.26	0.16	0.19	0.16	0.28
Change (%) ^1^	7.92%	−58.99%	36.67%	1.77%	23.97%	−59.60%

^1^ Refers to the average control treatment where only 5% cement was used (for clay average from 17 samples and for sand average from 19 samples).

**Table 10:** Selected hydraulic (1–3) and mechanical parameters (4–5) for the control scenarios (only soil + 5% cement).

**Additive**	**+5% Cement (Control Scenario)**	
**Soil**	**Sand**	
1. Frost resistance	%	35.6
2. Capillary rise (4 h)	g H_2_O/g soil dm (%)	6.0
3. Water absorption (24 h)	g H_2_O/g soil dm (%)	9.9
4. Compressive strength 7 d	MPa	1.150
5. Compressive strength 28 d	MPa	1.983
**Additive**	**+5% Cement (Control Scenario)**	
**Soil**	**Clay**	
1. Frost resistance	%	NA *
2. Capillary rise (4 h)	g H_2_O/g soil dm (%)	4.24
3. Water absorption (24 h)	g H_2_O/g soil dm (%)	11.5
4. Compressive strength 7 d	MPa	0.705
5. Compressive strength 28 d	MPa	0.844

* Not Available—the samples fell apart as they did not maintain their solid structure after 14 cycles of freezing.

**Table 11:** Selected hydraulic (1–3) and mechanical parameters (4–5) for the best waste-based additives for sandy soil, sorted in relation to frost resistance. The values of properties 4 and 5 are presented as changes (%) in relation to the control scenario value (with cement addition only). The control values were as follows: 1:35.6%, 2:6.0%, and 3:9.9%.

Additive	A EPW from WPEF	A + NaOH	Waste Tires Pyrolytic Oil WTPO (em.)	A + Waste H_2_SO_4_	A + “By-Pass” Ash + NaOH	“By-Pass” Ash + MgO
Soil	Sand					
1. Frost resistance	93.03%	87.58%	74.31%	69.45%	57.25%	48.47%
2. Capillary rise (4 h)	2.31%	6.69%	2.45%	1.43%	3.46%	6.55%
3. Water absorption (24 h)	7.93%	8.07%	9.25%	6.63%	6.26%	10.55%
4. Compressive strength ^1^	15.10%	33.03%	17.47%	37.46%	69.51%	47.14%
5. Compressive strength ^2^	−3.16%	23.70%	18.88%	6.29%	24.71%	4.11%

^1^ Relative change after 7 days (%); ^2^ relative change after 28 days (%).

**Table 12:** Selected hydraulic (1–3) and mechanical parameters (4–5) for the best waste-based additives to sandy soil. The results were sorted, taking into account the sealing properties described by capillary rise. The values of properties 4 and 5 are presented as a % of the control scenario value (with cement addition only). The control values were as follows: 1:35.6%, 2:6.0%, and 3:9.9%.

Additive	A EPW from WPEF	EPW from WMP + SS ^3^	B Sulfonated EPW from WMP	B + Nivea soap Waste	B + Cream Waste	A + Waste H_2_SO_4_
Soil	Sand					
1. Frost resistance	93.03%	NA	NA	NA	NA	69.45%
2. Capillary rise (4 h)	2.31%	0.45%	0.55%	0.96%	1.11%	1.43%
3. Water absorption (24 h)	7.93%	1.98%	5.63%	5.14%	5.05%	6.63%
4. Compressive strength ^1^	15.10%	41.02%	31.64%	26.62%	37.03%	37.46%
5. Compressive strength ^2^	−3.16%	NA	NA	NA	NA	6.29%

^1^ Relative change after 7 days (%); ^2^ relative change after 28 days (%); ^3^ sodium stearate (C_18_H_35_NaO_2_).

**Table 13:** Selected hydraulic (1–3) and mechanical parameters (4–5) for the best waste-based additives for clay. The results were sorted, taking into account the sealing properties described by capillary rise. The values of properties 4 and 5 are presented as a % of the control scenario value (with cement addition only). The control values were as follows: 2:4.24% and 3:11.5%.

Additive	A EPW from WPEF	A + NaOH + TequatLC90i	A + “By-Pass” Waste Ash + NaOH	A + “By-Pass” Waste Ash + TequatLC90i	A + Waste H_2_SO_4_	A + NaOH
Soil	Clay					
1. Frost resistance	NA	NA	NA	NA	NA	7.53%
2. Capillary rise (4 h)	6.92%	4.18%	4.06%	3.61%	2.82%	2.36%
3. Water absorption (24 h)	13.71%	11.38%	11.56%	11.51%	9.36%	8.38%
4. Compressive strength ^1^	−31.84%	−5.92%	−2.43%	−24.73%	80.68%	40.84%
5. Compressive strength ^2^	−40.71%	NA	NA	NA	64.42%	119.07%

^1^ Relative change after 7 days (%); ^2^ relative change after 28 days (%).

**Table 14:** Selected hydraulic (1–3) and mechanical parameters (4–5) for the best waste-based additives for sandy soil. The results are sorted, taking into account compressive strength after 7 days. The values of properties 4 and 5 are presented as a % of the control scenario value (with cement addition only). The control values were as follows: 1:35.6%, 2:6.0%, and 3:9.9%.

Additive	A EPW from WPEF	A + Waste H_2_SO_4_	EPW from WMP + “By-Pass” Waste Ash + NaOH	Sulfonated EPW from WMP	EPW from WMP + Cream	EPW from WMP + Cream + Powder
Soil	Sand					
1. Frost resistance	93.03%	69.45%	57,25%	NA	NA	NA
2. Capillary rise (4 h)	2.31%	1.43%	3.46%	0.99%	0.98%	0.95%
3. Water absorption (24 h)	7.93%	6.63%	6.26%	3.91%	4.31%	2.81%
4. Compressive strength ^1^	15.10%	37.46%	79.60%	88.31%	101.42%	142.42%
5. Compressive strength ^2^	−3.16%	6.29%	27.59%	NA	NA	NA

^1^ Relative change after 7 days (%); ^2^ relative change after 28 days (%).

**Table 15:** Selected hydraulic (1–3) and mechanical parameters (4–5) for the best waste-based additives for clay. The values of properties 4 and 5 are presented as a % of the control scenario value (with cement addition only). The control values were as follows: 2:4.24% and 3:11.5%.

Additive	A EPW from WPEF	A + NaOH	A + NaOH + Chewing Gum (em.)	Chewing Gum (em.) + NaOH + “By-Pass” Waste Ash	A + NaOH + TequatLC90i	A + ProRoad Waterproof
Soil	Clay					
1. Frost resistance	NA	7.53%	NA	NA	NA	NA
2. Capillary rise (4 h)	6.92%	2.36%	10.21%	9.98%	0.98%	16.42%
3. Water absorption (24 h)	13.71%	8.38%	NA	NA	4.31%	NA
4. Compressive strength ^1^	−31.84%	40.84%	50.30%	23.25%	101.42%	11.67%
5. Compressive strength ^2^	−40.71%	119.07%	NA	NA	NA	−11.37%

^1^ Relative change after 7 days (%); ^2^ relative change after 28 days (%).

**Table 16:** Selected hydraulic (1–3) and mechanical parameters (4–5) for the best waste-based additives for sandy soil. The values of properties 4 and 5 are presented as a % of the control scenario value (with cement addition only). The control values were as follows: 1:35.6%, 2:6.0%, and 3:9.9%.

Additive	A EPW from WPEF	A + Waste H_2_SO_4_	“By-Pass” Waste Ash + MgO	WTPO (em.)	A + NaOH	EPW from WMP +“By-Pass” Ash + NaOH
Soil	Sand					
1. Frost resistance	93.03%	69.45%	48.47%	74.31%	87.58%	57.25%
2. Capillary rise (4 h)	2.31%	1.43%	6.55%	2.45%	6.69%	3.46%
3. Water absorption (24 h)	7.93%	6.63%	10.55%	9.25%	8.07%	6.26%
4. Compressive strength ^1^	15.10%	37.46%	55.90%	17.47%	40.95%	79.60%
5. Compressive strength ^2^	−3.16%	6.29%	6.52%	18.88%	26.57%	27.59%

^1^ Relative change after 7 days (%), ^2^ Relative change after 28 days (%).

**Table 17:** Selected hydraulic (1–3) and mechanical parameters (4–5) for the best waste-based additives clay. The values of properties 4 and 5 presented as a % of the control scenario value (with cement addition only). The control values were as follows: 2:4.24% and 3:11.5%.

Additive	A EPW from WPEF	A + ProRoad Waterproof	A + Waste H_2_SO_4_	A + NaOH
Soil	Clay			
1. Frost resistance	NA	NA	NA	7.53%
2. Capillary rise (4 h)	6.92%	16.42%	2.82%	2.36%
3. Water absorption (24 h)	13.71%	NA	9.36%	8.38%
4. Compressive strength ^1^	−31.84%	11.67%	80.68%	40.84%
5. Compressive strength ^2^	−40.71%	−11.37%	64.42%	119.07%

^1^ Relative change after 7 days (%), ^2^ relative change after 28 days (%).

**Table 18:** Selected hydraulic (1–3) and mechanical parameters (4–5) for the best waste-based additives for sandy soil, sorted using optimization parameter (6). The control values were as follows: 1:35.6%, 2:6.0%, and 3:9.9%.

Additive	A EPW from WPEF	A + Waste H_2_SO_4_	WTPO (em.)	WMP + “By-Pass” Waste Ash + NaOH	A + NaOH
Soil	Sand				
1. Frost resistance	93.03%	69.45%	74.31%	57.25%	87.58%
2. Capillary rise (4 h)	2.31%	1.43%	2.45%	3.46%	6.69%
3. Water absorption (24 h)	7.93%	6.63%	9.25%	6.26%	8.07%
4. Compressive strength ^1^	15.10%	45.63%	17.47%	79.60%	40.95%
5. Compressive strength ^2^	−3.16%	8.75%	18.88%	27.59%	26.57%
6. Optimization parameter ^3^	67.5	12.90	19.49	26.31	49.68

^1^ Relative change after 7 days (%); ^2^ relative change after 28 days (%); ^3^ expressed as a ratio between capillary force (% H2O/% soil d.m. divided by 28 days compressive strength in MPa).

**Table 19:** Selected hydraulic (1–3) and mechanical parameters (4–5) for the best waste-based additives for clay soil; sorted using the optimization parameter (6). The control values were as follows: 1:35.6%, 2:4.24%, and 3:11.5%.

Additive	A EPW from WPEF	A + Waste H_2_SO_4_	A + NaOH
Soil	Clay		
1. Frost resistance	6.92%	NA	7.53%
2. Capillary rise (4 h)	13.71%	2.82%	2.36%
3. Water absorption (24 h)	−31.84%	9.36%	8.38%
4. Compressive strength ^1^	−40.71%	80.68%	40.84%
5. Compressive strength ^2^	6.92%	64.42%	119.07%
6. Optimization parameter ^3^	231.49	37.68	24.32

^1^ Relative change after 7 days (%); ^2^ relative change after 28 days (%); ^3^ expressed as a ratio between capillary force (% H2O/% soil d.m. divided by 28 days compressive strength in MPa).

## Data Availability

Data are contained within the article.
